# Renewal of spatial response distributions in rats

**DOI:** 10.1002/jeab.70145

**Published:** 2026-09-20

**Authors:** Rodrigo Benavides, Brissa Gutiérrez, Rogelio Escobar, Carlos Flores

**Affiliations:** ^1^ Universidad Nacional Autónoma de México México; ^2^ Universidad de Guadalajara México

**Keywords:** extinction‐induced variability, operant renewal, rats, response generalization, response location

## Abstract

Operant renewal refers to the relapse of eliminated behavior as a function of context change. Two experiments were performed to assess operant renewal effects in rat subjects with different response location criteria. In both experiments, subjects were trained to respond to a variable‐interval schedule on an array of five levers. For Experiment 1, in Context A, only responses on the middle lever were reinforced. For these subjects, response distributions formed a generalization gradient around the middle lever. For Experiment 2, responses on any lever could meet the reinforcement criteria. In this case, the response distribution across levers varied between subjects but was consistent within subjects across time. During both experiments, responding was next extinguished in Context B, which increased variability in response distributions. The reintroduction of Context A decreased variability levels. For most rats in Experiment 1 and some rats in Experiment 2, the pattern of responding during testing was more similar to the pattern during training than to the pattern at the end of extinction. The results suggest that renewed response distributions across locations varied as a function of the initial training conditions*.*

Extinction procedures hold significant clinical relevance, as response reduction is the objective of many behavioral interventions; however, responses reduced through extinction may recover when conditions change, limiting the long‐term effectiveness and generality of extinction‐based interventions (Kimball, Greer, et al., 2023; Lattal et al., 2013). Because of this, response recovery after extinction has been widely studied in basic and translational research literature with animal models of relapse following treatment (Bouton, 2002; Podlesnik et al., 2017, 2023). Studies concerned with this line of research usually employ three‐phase procedures in which responses are sequentially reinforced, extinguished, and then tested for recurrence. In such procedures, recurrence is typically evaluated as an increase in response rate during the test phase, usually while extinction remains in effect. Aside from response rate, less attention has been directed toward different dimensions of relapsed responses.

Response location is a dimension of behavior usually neglected by empirical studies (Kono, 2017), especially in the literature concerning response recurrence. However, a better understanding of the determinants of response location could represent a valuable complement to frequency measures like response rate. Research has demonstrated that reinforcement schedules regulate not only when organisms respond but also where responses occur, with systematic effects on spatial variability and shifts in location across conditions (e.g., Eckerman & Lanson, 1969; Kono & Tanno, 2020). Investigating response location in basic experimental settings allows researchers to identify how environmental contingencies shape topographical features of behavior, offering a more comprehensive understanding of operant units beyond their frequency.

Reinforcement of a specific response dimension, such as response location, tends to increase the frequency of similar responses, even if they have not been directly reinforced. This collateral effect of reinforcement is typically regarded as response generalization (Catania, 1973) and has been described as generalization gradients when the distribution of responses across some dimension is examined as a function of their similarity to a previously reinforced value. In such cases, responding is typically more frequent at or near the trained value and decreases systematically as similarity decreases, yielding an orderly, graded function. This process has been replicated across a wide range of response dimensions and is arguably a basic behavioral process that allows for response variability (Skinner, 1938). It is important not to conflate response generalization with stimulus generalization, as the latter refers to systematic changes in responding as a function of variation in antecedent stimuli correlated with different reinforcement histories (e.g., Okouchi, 2003; Okouchi et al., 2021). In the present article, we focus on response generalization, in which reinforcement for one response increases the likelihood of related responses.

Research on response generalization typically employs location as the dimension of analysis. For example, Escobar and Bruner (2007) used an experimental chamber with an array of seven levers aligned on the front panel in which only presses on the central lever were reinforced. The authors observed that the rats not only pressed the reinforced lever but also engaged in responding on the other levers, with response frequency decreasing as the distance from the central lever increased.

In one of the few studies analyzing relapse of response generalization, Diaz et al. (2019) observed the resurgence of generalization gradients. In a resurgence procedure, subjects acquire a target response, which is then disrupted during the training of an alternative response. Finally, introduction of an extinction procedure during the last phase tends to increase the frequency of the originally trained target behavior. In the procedure by Diaz et al., rats were trained to respond to the central lever of an array of seven levers. During the first phase, a generalization gradient was observed such that responses were most frequent on the central lever, with decreasing frequencies farther away from the center. Afterward, during the second phase, responses on the lever were extinguished while chain pulling was reinforced (alternative response). During the third and final phase, all responses were exposed to extinction, during which lever pressing transiently increased in frequency. The authors noted that the distribution of responses across the levers during the final phase of the procedure was similar to the one observed during training. This suggests that the increase in responding induced by the extinction procedure is not random but constrained by previous exposure to reinforcement contingencies.

A related phenomena in the recurrence literature is response renewal, in which changes to contextual cues renew responding. Therefore, a renewal effect refers to increases in previously extinguished responding when the stimulus or contextual conditions during extinction change (Bouton et al., 2011). For example, Nakajima et al., (2000) trained rats to lever press in Context A consisting of an experimental chamber with a flashing stimulus light, a chain suspended from the ceiling, and a black acrylic plate covering the floor. Afterward, some of the rats were exposed to an extinction procedure in the same context, whereas other rats experienced extinction in a different Context B. This alternative context was characterized by the absence of the chain and black acrylic, the stimulus light on continuously, and a smaller walking area, with the floor restricted by a white acrylic plate tilted toward the back panel. As would be expected under extinction conditions, all rats ceased responding after a few sessions. However, rats that experienced extinction in the alternative Context B showed a transient increase in lever pressing when exposed again to Context A, even with extinction still in effect.

Renewal studies have been especially relevant to translational research, as changes in contextual stimulation across experimental phases have been described as a useful analogue to changes during treatment situations (Kelley et al., 2015; Mace & Critchfield, 2010; Muething et al., 2020). In these studies, the focus is often directed toward ways of mitigating (Kimball, Greer, et al., 2023) or increasing (King et al., 2024) the frequency of relapsed behavior during the final phase of the procedure. As with resurgence procedures, little attention has been given to relapse of different dimensions of responses in renewal studies. Even though there are some reports regarding the renewal of response topography (e.g., Todd, 2013), to our knowledge, there are no current reports analyzing the renewal of generalization gradients.

Although similar to resurgence procedures in the sequential structure of three phases, renewal procedures differ in a way that merits attention. Usually, during the second phase of a renewal procedure, no alternative response is reinforced (see Kimball et al., 2020; Kimball, Salvetti, et al., 2023, for some exceptions). This difference allows induction effects to develop from the withdrawal of the reinforcement contingency of the previous phase (Baum, 2018; Podlesnik & Baum, 2024). It should be noted that although the terms response induction and response generalization have sometimes been used interchangeably (Catania, 1973), in the present article response induction will refer exclusively to increases or changes in the allocation of responses produced by the worsening or withdrawal of reinforcement, as when extinction increases responding at previously infrequent locations or increases response variability.

In a now‐considered‐classic study about extinction‐induced variability, Antonitis (1951) studied the variability of response location across a horizontal slot in an experimental chamber. During his experiment, rats were trained on a response chain in which they (a) left the feeding compartment, (b) made one or more nose‐poke responses anywhere along a 50‐cm response slot, and (c) returned to the feeding compartment, where food was delivered after completion of the chain. As sessions progressed, the author reported decreases in the mean variation from the median distance between nose‐poke responses, meaning that the variation in response locations decreased after continuous reinforcement conditions. In the following phase, Antonitis exposed rats to an extinction condition, during which median distance between responses increased. Ensuing phases with a reversal design showed that response location tended to vary as a function of reinforcement availability; when reinforcement was present, responses tended toward the same location, and when extinction was reintroduced, response locations tended to vary.

In summary, the findings of Diaz et al. (2019) show that generalization gradients form and relapse under resurgence procedures. We also know from the work of Antonitis (1951) and many other authors since (Eckerman & Lanson, 1969; Grunow & Neuringer, 2002; Iversen, 2017) that extinction procedures induce variability in responding. Therefore, by including a specific period for response extinction, renewal procedures allow for an interesting assessment of behavioral phenomena during the different phases of the procedure: reinforcement (formation of generalization gradients), extinction (extinction‐induced variability), and testing (renewal of generalization gradients). If renewal procedures are to be useful as a model to understand relapse, it is paramount to describe in greater detail how response patterns shift during different phases of the procedure.

Analyzing behavioral patterns during extinction and relapse could yield useful insights for applied practices, as changes in context following treatment have been shown to renew previously extinguished problem behavior (Muething et al., 2020; Saini, et al., 2018). Thus, the purpose of the present study was to (a) describe how generalization gradients that develop during reinforcement change when exposed to the extinction phase of a renewal procedure and (b) evaluate whether these gradients are susceptible to renewal during the final phase of the procedure. We made three specific predictions. First, during reinforcement, responding would organize around the reinforced location, producing a differentiated generalization gradient centered on that location. Second, during extinction, the gradient would degrade (i.e., become flatter and/or shift), reflecting extinction‐induced variability. Third, if relapse occurred during the final phase of the renewal procedure, the organization of responding would reemerge such that the gradient would recover characteristics observed during reinforcement (e.g., increased differentiation around the previously reinforced location). To assess these hypotheses, we carried out Experiment 1.

## EXPERIMENT 1

### Method

#### Subjects

Six experimentally naive male Wistar rats approximately 9 months old served as subjects. The rats were housed individually in a room maintained on a 12:12 light–dark cycle, with lights on at 7:00 a.m. Experimental sessions were conducted during the light portion of the cycle at 1200 hours every day. Rats were maintained at 85% of their ad libitum weight, with free access to water. Supplemental feeding was provided 1 hr after the experimental session to maintain target body weight. The care and housing of the subjects complied with the animal care guidelines of the institution.

#### Apparatus

Three 30‐ × 20‐ × 20‐cm operant chambers were built by means of 3D printing (Escobar et al., 2022). One chamber was used for pretraining and one for each experimental context: Context A and B. The pretraining chamber was placed on an open rack, without a sound‐dampening cubicle. It was equipped with a 4‐ × 2‐cm response lever centered at 5 cm from the floor on the front wall. The chamber also had a stimulus light above the lever, but it was never turned on. No particular smell was arranged for this chamber, and the acrylic panels were left transparent. It was also equipped with a receded food receptacle on the back wall, where a pump delivered .10 ml of 30% condensed milk solution. The same condensed milk solution was employed during all reinforcement conditions in all chambers, but it was not present during extinction or testing. Both experimental context chambers were placed in sound‐dampened cubicles and were equipped with five 1‐ × 2‐cm levers mounted 2 cm apart from each other in the horizontal axis of the front wall, at 5 cm from the floor, with two stimulus lights above them positioned on the left and right side of the wall, respectively. The back wall was equipped with a receded food receptacle for condensed milk deliveries. A houselight positioned above the food receptacle was used for general illumination and remained on throughout all phases of the experiment. One context was characterized by the presence of white horizontal lines across the chamber's acrylic panels, a black ceiling, red stimulus lights, 60 dB of white noise, and the smell of flowers. The other context was characterized by transparent acrylic panels and ceiling, white stimulus lights, no white noise, and the smell of citrus. The smells were achieved by placing Great Value Scented Gel Fresheners inside the sound dampened cubicles. The fresheners were replaced weekly during Phase 1 and during the first day of exposure to Phase 2 and Phase 3, respectively. Experimental events and response registry were managed by the Arduino‐Visual Basic interface (Escobar & Perez‐Herrera, 2015).

#### Procedure

##### Pretraining

Subjects were trained to lever press in the pretraining chamber by differential reinforcement of successive approximations for two sessions. Afterward, lever pressing was reinforced until 50 condensed milk deliveries were obtained in less than 30 min for two consecutive sessions under a continuous reinforcement schedule. After that, a variable‐interval (VI) schedule of reinforcement was increased from 5 s to 10, 15, and 20 s following Fleshler and Hoffman's (1962) progression with 10 iterations without replacement. The same criteria of 50 reinforcers in less than 30 min during two sessions was required for each increment. The number of pretraining sessions needed to reach the final VI criterion varied for each rat, ranging from eight to 13 sessions. Two sessions were conducted per day throughout pretraining. Sessions were always carried out both during pretraining and the experimental phases 7 days a week.

##### Phase 1: Training in Context A

Subjects were exposed to one of the chambers with five levers. From this point forward, sessions were conducted once per day such that contextual changes were experienced across days rather than within the same day. Exposure to the contexts was counterbalanced such that half of the rats were exposed to each of the two available contexts as the training context. Lever pressing was reinforced according to a VI 20‐s schedule, but only on the middle lever, to allow generalization effects to develop in both directions. To facilitate discrimination between the levers during training, all responses to the middle lever were signaled by a .5‐s activation of the stimulus lights as response feedback. Responses on other levers were registered but had no programmed consequences. Sessions ended after 50 reinforcer deliveries or 30 min, whichever occurred first. This phase was in effect for 15 sessions.

##### Phase 2: Extinction in Context B

Subjects were exposed to the alternative context depending on their counterbalance status, and all reinforcement was withheld (i.e., extinction). Responses during this phase were recorded but had no programmed consequences aside from the activation of the stimulus light for presses on the middle lever as in the previous phase, which was always held constant. This phase was in effect for six sessions.

##### Phase 3: Renewal test in Context A

Subjects were exposed to their respective original training context. Responses on every lever were again recorded but remained on extinction as in the previous phase such that the only consequence was the activation of the stimulus light when pressing the middle lever. This phase was in effect for six sessions.

### Results

**FIGURE 1:**
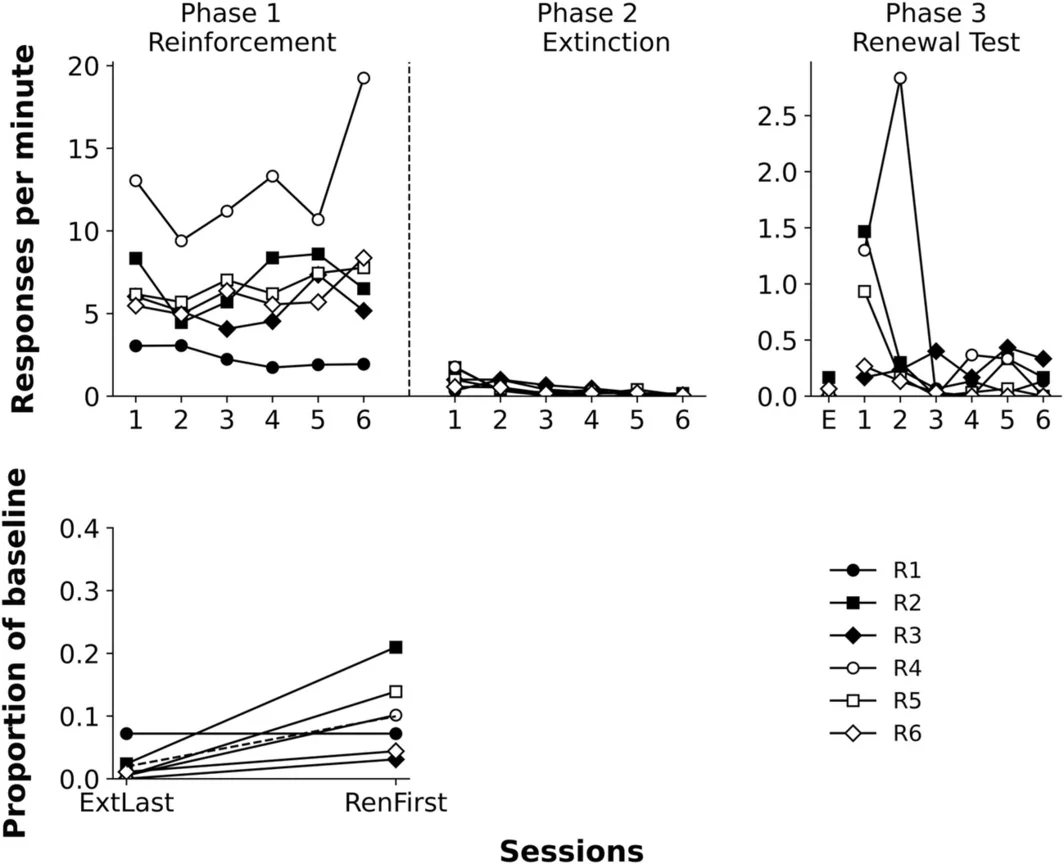
Responses per minute at the active lever across Experiment 1. Please note the different *y*‐axis for Phase 3. Session “E” in Phase 3 represents responses from the last extinction session. Baseline for the lower panel was the mean of the last six sessions of Phase 1.

The upper panels of Figure 1 show responses per minute on the reinforced lever across experimental phases for Rats R1 through R6. During Phase 1, response rates varied widely across subjects ranging 3–20 responses per minute. During extinction in Phase 2, response rates steadily decreased, reaching values below 0.5 responses per minute for most subjects by the sixth session. Notice that for Phase 3 the panel shows a reduced *y*‐axis to better highlight increases in responding. The first segment of that panel shows responses during the last extinction session, during which data points from R1 and R2 overlap at 0.16 and R3 stays at 0 responses per minute. For all subjects but R1, an increase in responding was observed during the first test session of Phase 3, especially for R2, R4, and R5. Both R1 and R4 showed a transient increase in responding during exposure to the second test session. After the first two test sessions, responding stabilized between 0 and 0.5 responses per minute for all subjects.

Given the small sample size, we opted for nonparametric statistical methods for comparisons across phases. All pairwise comparisons for the dependent variables were conducted using the Wilcoxon signed‐rank test, which is appropriate for analyzing dependent samples without assuming normality. The test was conducted to compare responses during the last extinction session and the first test session. Results failed to identify a significant difference between conditions (*W* = 1, *Z* = −1.99, *p* = .063). However, given that for some subjects most responding during testing occurred at Test Session 2, we repeated the test considering the mean of the last two extinction sessions and the mean of the first two test sessions, which revealed a significant difference between conditions (*W* = 0, *Z* = −2.20, *p* = .031).

As an additional measure of difference between Phase 2 and Phase 3 responding, the lower panel shows responses during the last Phase 2 session (ExtLast) and the first Phase 3 session (RenFirst) as a proportion of baseline. The proportion of baseline analysis was included to normalize renewal effects relative to each subject's own Phase 1 performance, controlling for individual differences in absolute response rates during the reinforcement phase. Proportion of baseline was calculated by dividing response rates in the last Phase 2 session and the first Phase 3 session by the average response rate from the last six Phase 1 sessions. The dotted line shows the average between the data points. Proportion of baseline rates of behavior increased from the end of Phase 2 to the beginning of Phase 3 for all subjects except R1. Note that a proportion of 1 would signal responding close to Phase 1 responding. For all rats, proportion of baseline responding stood between .05 and .25.

**FIGURE 2:**
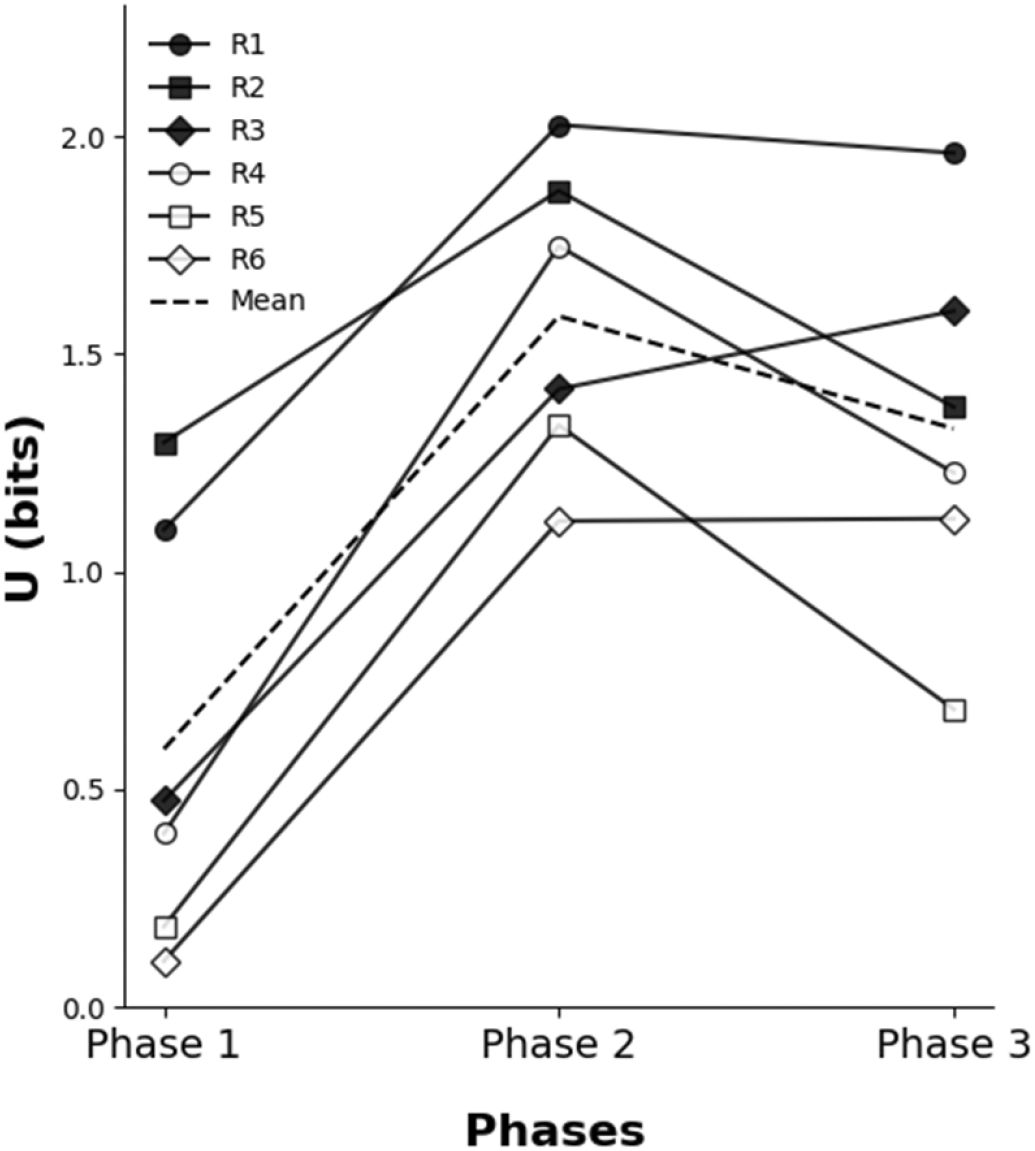
Pooled *U* values calculated with total responses from the last six sessions of each phase for Experiment 1.

Figure 2 shows pooled *U* values across experimental phases as a measure of variation in response distributions. *U* values are measured in bits and represent the variability in responding. It is a ubiquitous measure in behavioral variability studies and proves useful when describing variation in response patterns, such as when responses are exposed to extinction procedures (Kong, et al., 2017). It is calculated as the sum of the probabilities of responding on each operanda multiplied by the logarithm of those probabilities (Johansen et al., 2007):

$$U=-\sum p\;\log_{2}\;\left(p\right)$$

Probabilities were calculated as relative frequencies based on the total number of responses emitted across the last six sessions of Phase 1 and across the six sessions of Phases 2 and 3, respectively. For example, if a rat emitted 120 responses in total during the six sessions of Phase 2 and 60 of those occurred on Lever 3, the relative frequency (probability) for Lever 3 in that phase would be .50. In this context, the *U* value ranges from 0 bits in the case that all responses occurred at the same lever (low variability) to 2.3 bits in the case that responding is evenly distributed across all five levers (high variability). Galizio et al. (2018) have shown that entropy‐based indices such as *U* are sensitive to sample size, so low response counts during extinction can artifactually depress or constrain estimates; consequently, pooling responses across sessions is one recommended way to stabilize *U*. The pooled *U* value was computed by summing responses across sessions within each phase for each rat and then calculating entropy across the five response options. Thus, sessions were collapsed within phase, yielding a single *U* value per rat per phase for direct comparisons across phases.

Figure 2 shows *U* values for all subjects during the experiment. The dashed line signals the average of data points during that phase. During Phase 1, *U* values remained between 0.1 and 1.3 bits. During Phase 2, *U* values increased markedly relative to Phase 1 for all subjects, ranging from 1.1 to 2 bits, an increase that was statistically significant (*W* = 0, *Z* = −2.20, *p* = .031). This increase in *U* values relative to the previous phase signals that subjects started to respond at a different proportion over levers with previously low or null response counts. Even when response rates were low due to extinction, responding occurred in a very different spatial distribution. During Phase 3, *U* values stayed relatively close to the ones from the previous phase. Inspection of individual data points reveals that *U* values for R2, R4, and R5 shifted toward Phase 1 values. However, the Wilcoxon test failed to find a significant difference between Phases 2 and 3 (*W* = 4, *Z* = −1.37, *p* = .218).

**FIGURE 3:**
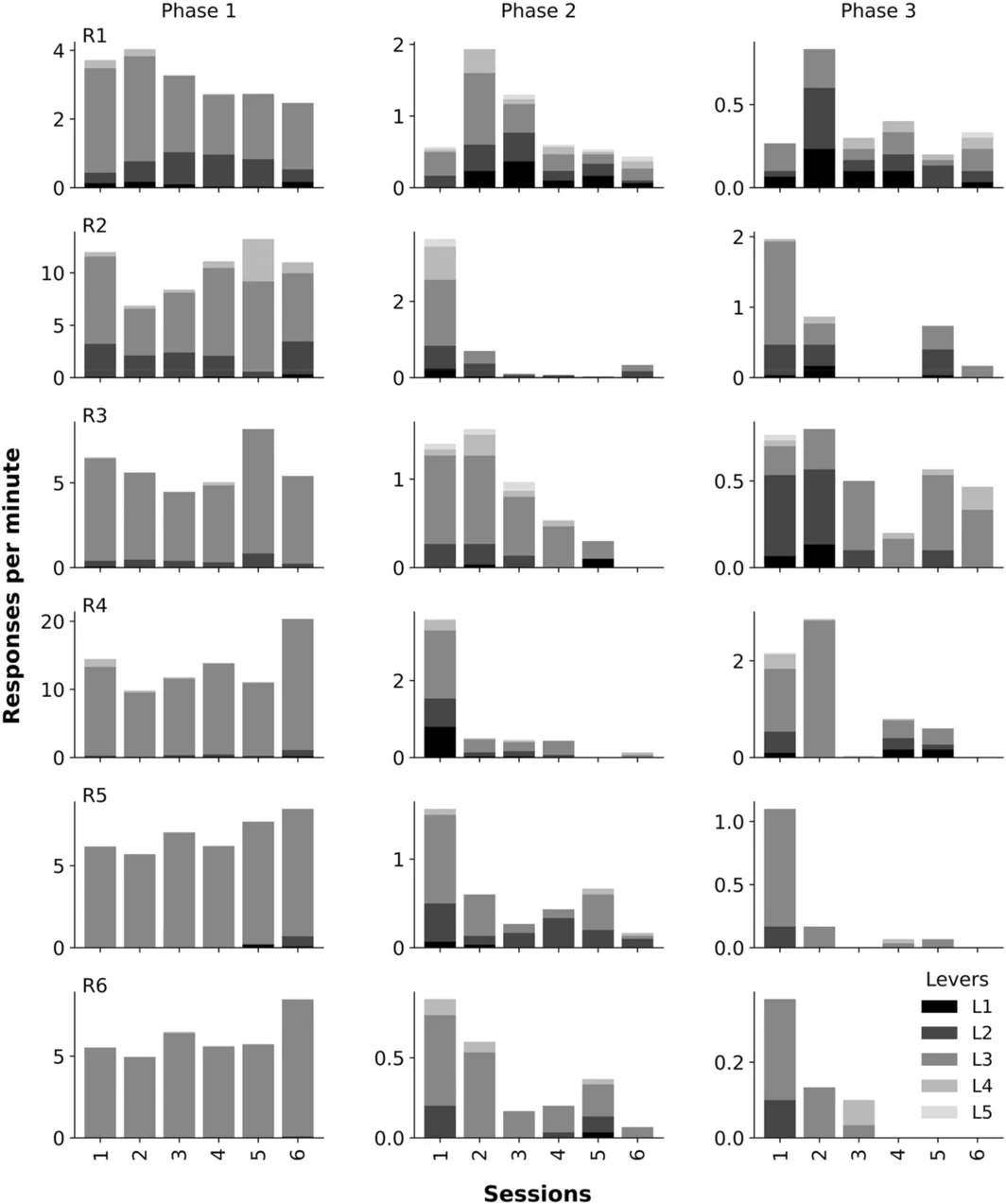
Response rates by lever across Experiment 1. Data from the last six sessions of Phase 1 and all sessions from Phases 2 and 3. Please note the individually scaled *y*‐axis for each rat and phase. Each color of the bars signals a different lever; the bigger the colored section, the bigger the proportion of responses at that particular lever.

Figure 3 shows responses per minute at each lever for all subjects during the last six sessions of Phase 1 and the six sessions of Phases 2 and 3. We chose a stacked bar approach to visually represent these data as a means to provide an intuitive description of spatial patterns at a glance. Each color of the bars signals a different lever; the bigger the colored section, the bigger the proportion of responses at that particular lever. Levers with no responses simply show no bar at the panels. During Phase 1, responding was mostly maintained at the middle lever (L3) for all subjects, as this was the only reinforced response option. For R1 and R2, responding at levers different from the middle one was somewhat frequent, but the middle lever was always the one with the higher response count. For R3 through R6, interaction with lateral levers was sparse. During Phase 2, responding progressively decreased for all rats. However, response distribution across the levers shifted. For example, R4 started to more evenly distribute responses across all levers during the first extinction session. Still, as sessions progressed, responses mostly went back toward the middle of the lever array. During Phase 3, response distributions continued to vary. However, for most subjects, an increase in responses allocated toward the middle lever was observed. During the first test session, R1, R2, R4, R5, and R6 responded both at the middle and the lateral levers that had more responses during the last reinforcement session. For R3, responses were also frequent on the previously reinforced lever during Test Sessions 3 through 6. For R6 in particular, responses remained frequent on the middle lever before ceasing altogether at Test Session 4. Overall, during the test phase, all subjects showed a tendency toward the middle lever.

**FIGURE 4:**
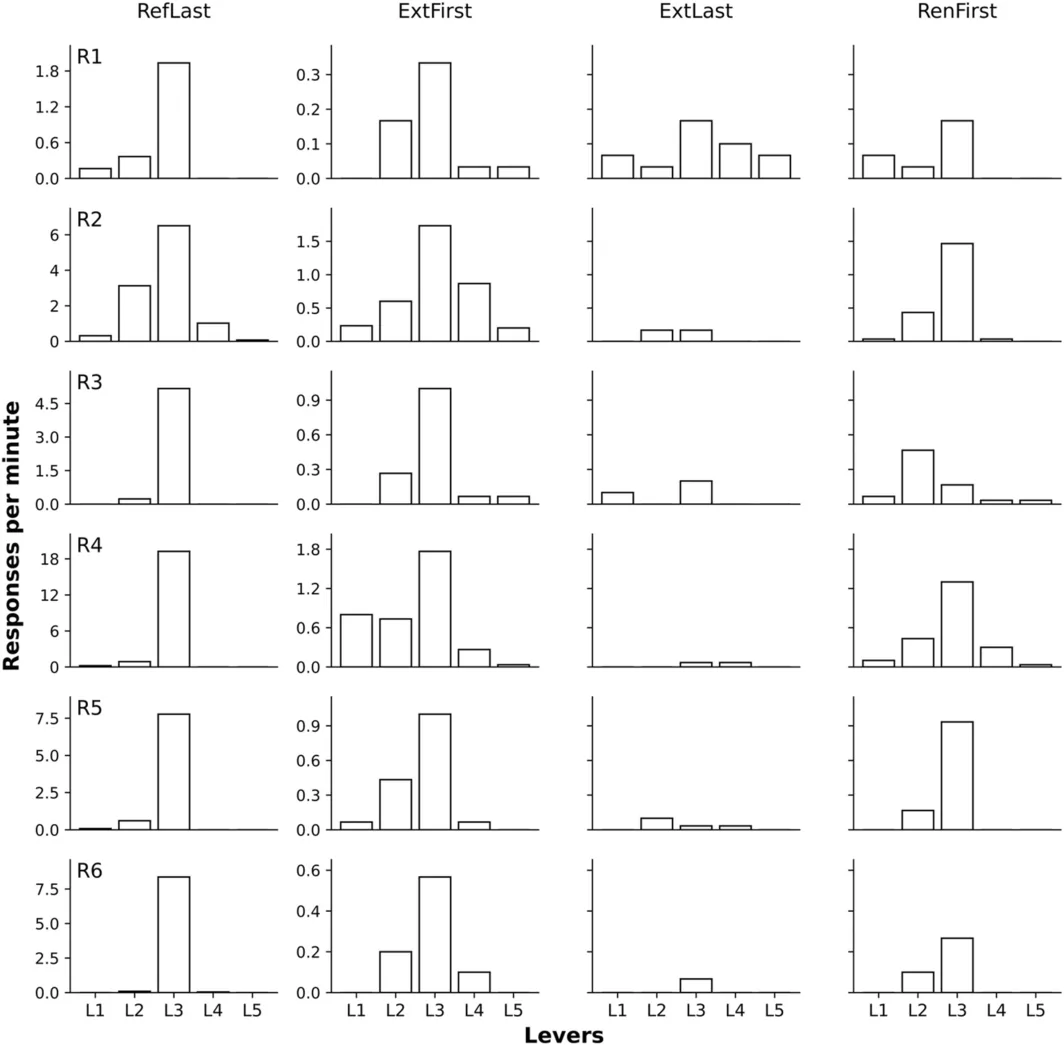
Responses per minute across levers for transition sessions in Experiment 1. RefLast = Last reinforcement session, ExtFirst = First extinction session, ExtLast = Last extinction session, and RenFirst = First renewal test session. The panel for R3 at ExtLast shows responses from the fifth extinction session, as no responses occurred during the last extinction session. Please note the different *y*‐axis between RefLast and the transition sessions under extinction.

To assess whether patterns of responding during reinforcement and testing were similar according to a quantitative criterion, we calculated Spearman's rank‐order correlations to compare the relative distribution of responses across levers for each subject. For each session, the total number of responses across all levers was calculated and responses on each lever were expressed as a proportion of that total, yielding a distribution of relative responding independent of overall rate. Spearman's rho was chosen because these proportional data are not guaranteed to meet assumptions of normality or linearity and the number of observations per distribution is limited to the number of levers. This nonparametric test is therefore well suited to evaluate the degree to which the rank ordering of lever preferences is preserved across phases. For example, if during the last session of Phase 1 a subject responded the most on L3, then L2, then L4, with little or no responding on L1 and L5 and the same rank order is observed at the beginning of another phase, the correlation approaches ρ = 1 even if the overall level of responding differs substantially. In contrast, if the relative ordering of levers changes (e.g., L2 records more responses than L3), the coefficient decreases. Thus, the correlations capture preservation of the spatial hierarchy of responding rather than changes in response frequency. Correlations were judged statistically significant at α = .05. Results are presented in Table 1. We suggest comparing the values from the table with Figure 4 for easier interpretation.

**TABLE 1:** Correlation between response distributions for Experiment 1.

Subject	RefLast‐ExtFirst	RefLast‐ExtLast	RefLast‐RenFirst	ExtFirst‐RenFirst	ExtLast‐RenFirst
R1	.684, *p* = .203	.132, *p* = .833	.895, *p* = .040*	.368, *p* = .542	.289, *p* = .636
R2	.900, *p* = .037*	.866, *p* = .058	.975, *p* = .005*	.821, *p* = .089	.889, *p* = .044*
R3	.918, *p* = .028*	.500, *p* = .391	.803, *p* = .102	.579, *p* = .306	.344, *p* = .570
R4	.872, *p* = .054	.148, *p* = .812	.821, *p* = .089	.700, *p* = .188	.577, *p* = .308
R5	.921, *p* = .026*	.487, *p* = .406	.918, *p* = .028*	.918, *p* = .028*	.648, *p* = .237
R6	1.000, *p* ≈ .000*	.725, *p* = .165	.918, *p* = .028*	.918, *p* = .028*	.790, *p* = .111

*Note*: Calculated with the distribution of responses between levers. RefLast = last session Phase 1. ExtFirst = first session Phase 2. ExtLast = last session Phase 2 with available data points. RenFirst = first session Phase 3.

* = statistically significant correlation. The first value of each cell shows ρ.

The RefLast–ExtFirst comparison shows generally high correlations (e.g., R2, R3, R5, and R6), indicating that at the onset of extinction the overall spatial hierarchy of responding was still largely preserved, despite the increase of responding on additional levers. In contrast, the RefLast–ExtLast comparison reveals that by the end of extinction correlations were markedly reduced for most subjects (e.g., R1, R3, R4, and R5), suggesting that the spatial pattern had reorganized and no longer resembled the reinforcement pattern. The RefLast–RenFirst comparison showed relatively high correlations for the majority of subjects (e.g., R1, R2, R5, and R6), indicating that the response hierarchy observed during reinforcement tended to reemerge during testing. The ExtFirst–RenFirst comparison yielded more variable and generally lower correlations, with significant correlations only for R5 and R6, suggesting that the renewal‐test distribution was not consistently similar to the distribution observed at the onset of extinction. To evaluate whether the renewal‐test distribution was instead more similar to the terminal extinction pattern, we also included the ExtLast–RenFirst comparison. These correlations were lower than the corresponding RefLast–RenFirst correlations for all subjects and were significant only for R2. Thus, the response distribution observed during testing appeared to resemble the distribution established during reinforcement more than the distributions observed either at the beginning or at the end of extinction.

Figure 4 shows individual generalization gradients for each rat across the four transition sessions analyzed in the previous correlations: the last session of Phase 1 (reinforcement), the first session of Phase 2 (onset of extinction), the last session of Phase 2, and the first session of Phase 3 (renewal test). Bars represent response rate on each lever (L1 through L5 from left to right). During the final session of Phase 1, responding was mainly concentrated on the trained lever (L3), with minimal responding on adjacent levers (L2 and L4) and near‐zero responding on L1 and L5 for most subjects (R2 was the only rat to show three responses per minute at L2). In the first session of Phase 2, overall response rates decreased substantially, although residual responding remained highest on L3, with near‐zero response rates at adjacent levers in most rats. By the final session of Phase 2, response rates were near zero across all levers for all rats. In the first session of Phase 3 (renewal test), responding reemerged primarily on L3 for all rats except R3, for which most responses occurred at L2, accompanied by lower levels of responding on adjacent levers.

Finally, we calculated obtained reinforcement rates during the last six sessions of Phase 1 by dividing obtained reinforcers by the duration of each session. Because reinforcement was arranged according to a VI 20‐s schedule, the theoretical maximum obtained reinforcement rate was approximately three reinforcers per minute. Mean reinforcement rates ranged from 1.31 to 2.28 reinforcers per minute across rats. Rats R2, R4, R5, and R6 obtained all 300 programmed reinforcers available across the last six Phase 1 sessions, with mean rates of 1.80, 2.28, 2.10, and 2.00 reinforcers per minute, respectively. R3 obtained 280 of 300 available reinforcers, with a mean rate of 1.58 reinforcers per minute, whereas R1 obtained 234 of 300 available reinforcers, with a mean rate of 1.31 reinforcers per minute.

### Discussion

The present experiment showed an increase in variability in response location after the shift from reinforcement to extinction. This can be interpreted as an instance of extinction‐induced variability, which is congruent with previous reports in the literature (Antonitis, 1951; Iversen, 2017; Santillán & Escobar, 2019). Even when response locations varied, the lever with the higher response frequency stayed the same, suggesting that the prior reinforcement contingency still exerted control over responding even after multiple sessions of extinction.

Following common definitions of relapse (e.g., Kuroda et al., 2020; Shvarts et al., 2020), we defined renewal as an increase in responding in Phase 3 relative to the last session of extinction. We observed a renewal effect for most subjects after the shift from the extinction context to the context previously correlated with reinforcement. This is also congruent with previous reports in the literature (Bouton et al., 2011; Nakajima et al., 2000). The renewal effect could mainly be observed for responses on the middle lever as well as near lateral levers (see Figures 3 and 4). Even though response rates were low, this can be interpreted as renewal of not only previously reinforced responses but also of response location and generalization gradients (Baum, 2018; Catania, 1973). For most rats, the highest number of responses during reinforcement and renewal occurred on the previously reinforced lever. Among the nonreinforced levers, the second‐highest number of responses tended to occur on one of the levers adjacent to the reinforced lever. That is, responding did not disperse evenly across all remaining options but was preferentially allocated to spatially contiguous alternatives. This systematic pattern is consistent with the development of a generalization gradient centered on the reinforced lever, even if the gradient was not perfectly symmetrical across all response locations. This finding is similar to that reported by Diaz et al. (2019) with a resurgence procedure and extends the generality of the renewal phenomena to responses maintained not by direct reinforcement but by generalization effects.

Finally, ExtFirst–RenFirst correlations were generally lower than RefLast–RenFirst correlations, with significant correlations observed for only two rats in the former comparison and for four rats in the latter. This difference suggests that the response distribution observed during testing was more similar to the distribution established during reinforcement than to the one observed at the onset of extinction. This distinction is important because responding during the renewal test also occurred under extinction and thus could be difficult to dissociate from extinction‐induced variability. However, the return to Context A did not appear to produce just a undirected redistribution of responding across levers. Rather, for most subjects, responding shifted toward the spatial organization previously established during reinforcement, especially toward the reinforced lever and adjacent response locations. Thus, the variability observed during extinction seems to reflect a reorganization of spatial responding produced by the withdrawal of reinforcement, whereas responding during testing reflected the renewal of features of a previously established response distribution. In this sense, the present data suggest that renewal and extinction‐induced variability were related but distinguishable processes.

## EXPERIMENT 2

In Experiment 1, reinforcement was delivered exclusively for responding at the middle lever, which facilitated the occurrence and measurement of generalization gradients. However, previous studies investigating extinction‐induced responses have often allowed subjects to freely vary their responses along a spatial continuum, typically across a horizontal axis (e.g., Antonitis, 1951; Ferraro & Branch, 1968; Eckerman & Lanson, 1969; Santillán & Escobar, 2019). Under these conditions, extinction‐induced variability becomes evident through increased dispersion of response locations when reinforcement is discontinued. To better align with the methods reported in such studies and explore induction effects during extinction more extensively, in Experiment 2, subjects were allowed to respond freely along the lever array, with reinforcement provided across all five response locations rather than only on the middle one. Thus, the purpose of this second experiment was to describe how stable response patterns maintained by reinforcement at different locations would degrade and recur when exposed to the different phases of a renewal procedure.

### Method

#### Subjects and apparatus

Six experimentally naive male Wistar rats approximately 9 months old served as subjects. Rats were maintained under the same conditions as Experiment 1 and also ran daily sessions at 1200 hours. The same three chambers were used. The gel fresheners were also replaced weekly, and the same condensed milk solution at 30% was used during Pretraining and Phase 1.

#### Procedure

##### Pretraining

Pretraining was carried out in the same fashion as Experiment 1. The number of sessions needed to reach the VI criterion ranged from 8 to 10 sessions. Again, pretraining sessions were carried out twice a day, and throughout all experimental phases, sessions were run 7 days a week.

##### Phase 1: Training in Context A

Rats were exposed to the chambers with five levers. Contexts were again counterbalanced such that half of the rats started in one context and the other three rats started the experiment in the alternative context. Responses on all levers could meet the VI criteria. To keep the methods between experiments as similar as possible, all responses on all levers now turned on the stimulus lights for 0.5 s to signal responses. Sessions ended after 50 milk deliveries or 30 min had elapsed. This phase was in effect for 15 sessions.

##### Phases 2 and 3: Extinction in Context B and renewal test in Context A

These phases were the same as during the previous experiment. During Phase 2, subjects were exposed to the alternative context, depending on their counterbalance status, and reinforcement was withheld. Responses during Phase 2 were recorded but had no programmed consequences aside from the activation of the stimulus lights. As for Phase 3, subjects were returned to their original training context with extinction still in effect. Again, all responses were recorded with the only consequence being the activation of the stimulus lights. Both phases were in effect during six sessions.

### Results

**FIGURE 5:**
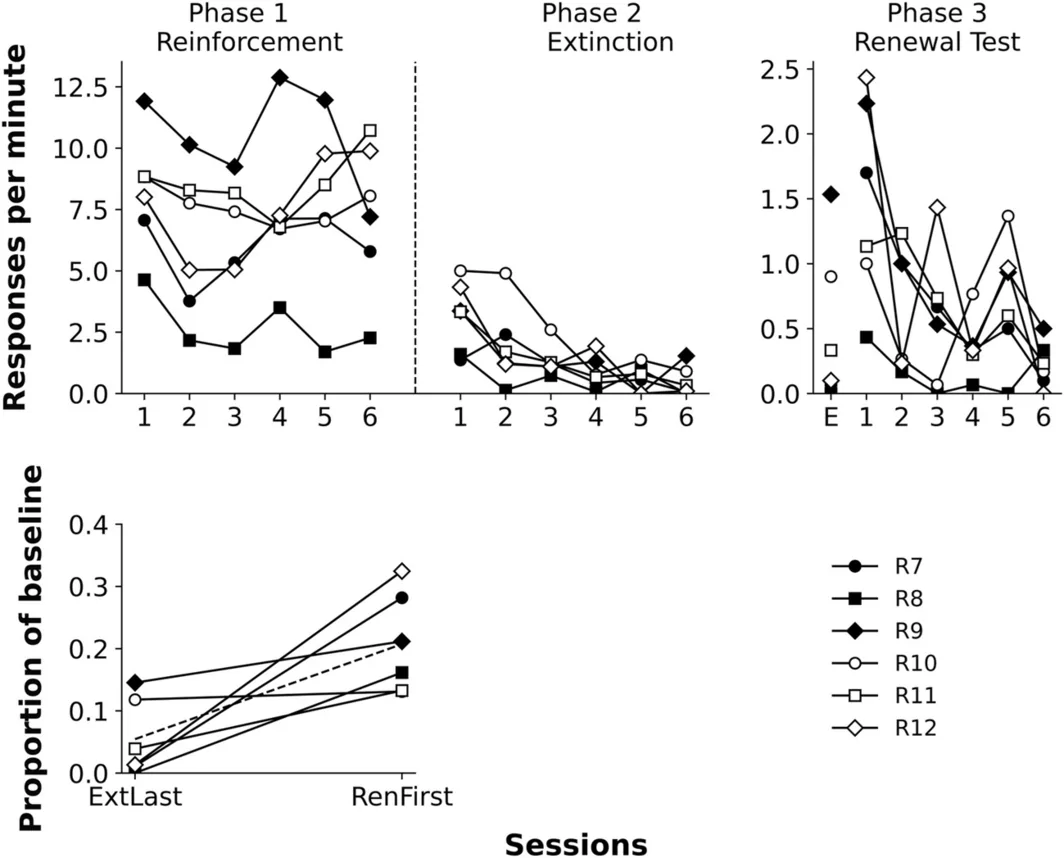
Responses per minute at all levers in Experiment 2. Please note the different *y*‐axis for Phase 3. Session “E” in Phase 3 represents responses from the last extinction session. Baseline for the lower panel was the mean of the last six sessions of Phase 1.

The upper panels of Figure 5 show responses per minute across experimental phases for rats R7 through R12. Responses per minute were calculated considering total responses on all levers divided by the total duration of the sessions. During Phase 1, response rates varied across subjects. Except for R8, which had a relatively low rate of responding, response rates ranged between 5 and 12 responses per minute. During Phase 2, when first exposed to extinction, response rates decreased for all subjects and stayed below 1 response per minute for most subjects by the last extinction session. During Phase 3, a transient increase in responding was observed for all subjects, especially during the first session, after which responding decreased to nearly 1 response per minute. A Wilcoxon rank‐sum test was conducted to compare responses during the last extinction session and the first test session. Results revealed a significant difference between conditions (*W* = 0, *Z* = −2.20, *p* = .031). The lower panel of Figure 5 shows responses as a proportion of baseline during the last Phase 2 session and the first Phase 3 session. For all subjects, responses increased relative to baseline. However, for R10 the increase was slight. Proportion of baseline responding stood between .15 and .40.

**FIGURE 6:**
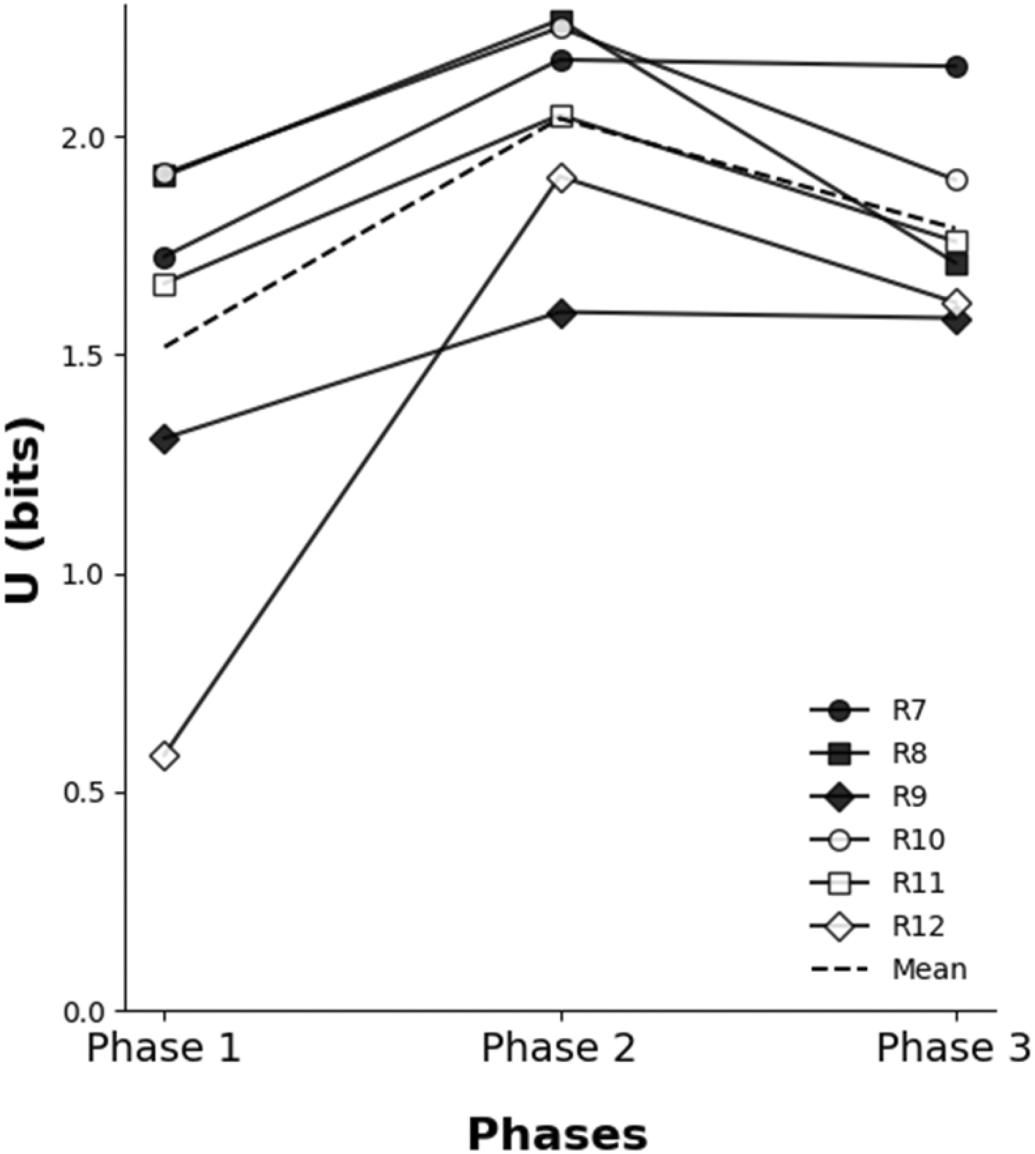
Pooled *U* values calculated with total responses from the last six sessions of each phase in Experiment 2.

Figure 6 shows *U* values across the experiment. During Phase 1, average *U* values remained relatively stable at around 1.5 bits for all rats except R12, indicating high variability in responding location. During Phase 2, *U* values increased for all subjects relative to the previous phase. The increase between Phase 1 and 2 was statistically significant (*W* = 0, *Z* = −2.20, *p* = .031). These *U* values of around 2 bits indicate patterns of responding in which most responses are evenly distributed across levers, approaching almost random distributions of responses. During Phase 3, U values shifted toward those observed during Phase 1 consistently for all rats. Although the magnitude of change was small for R7 (2.17 to 2.16) and R9 (1.60 to 1.58), the Wilcoxon test indicated that the shift in *U* value between Phases 2 and 3 was significant (*W* = 0, *Z* = −2.20, *p* = .031).

**FIGURE 7:**
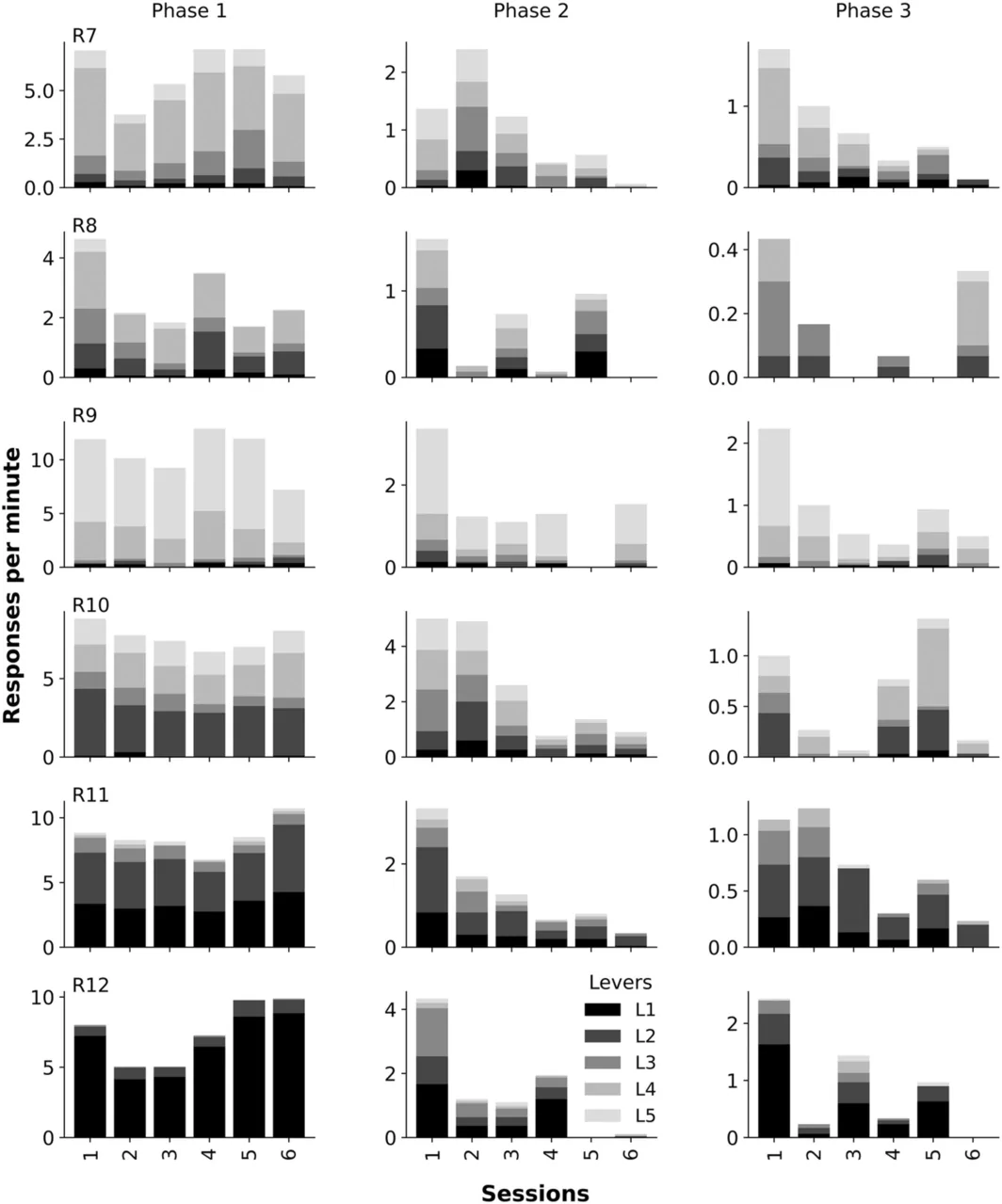
Response rates by lever across Experiment 2. Data from the last six sessions of Phase 1 and all sessions from Phases 2 and 3. Please note the individually scaled *y*‐axis for each rat and phase. Each color of the bars signals a different lever; the bigger the colored section, the bigger the proportion of responses at that particular lever.

Figure 7 shows responses per minute for R7 through R12 across levers during the last six sessions of Phase 1 and the six sessions of Phases 2 and 3. During Phase 1, even though response distributions varied for all subjects, stable patterns of responding developed through the sessions. For R7, R9, and R12, responses were mostly allocated toward one main lever (L4 for R7, L5 for R9, and L1 for R12), but many responses also occurred across the other levers for R7 and R9. R10 mostly distributed responses between two levers (L2 and L4), as well as R11 (L1 and L2), with few responses on other levers. Finally, R12 was the only rat to allocate most responses to a single lever (L1), with few responses to the lever next to it and no responses over the other three levers. During Phase 2, upon contacting the extinction procedure, response distribution across the levers shifted markedly for most rats. Except for R9, which kept responding at L5 throughout extinction, responses were more evenly distributed across all levers than during the previous phase for all rats. During Phase 3, response distributions continued to vary. For R7, R9, R10, R11, and R12, the lever with most responses during Phase 1 was also the lever with more allocated responses during the first Phase 3 session.

Results from the Spearman's rank‐order correlation test are presented in Table 2. The RefLast–ExtFirst correlations were generally moderate to high for most subjects (R7, R8, R9, R11, and R12), indicating that at the onset of extinction the spatial hierarchy of responding still resembled that established during reinforcement; R10 was the only subject showing little correspondence. The RefLast–ExtLast comparison, however, showed a more heterogeneous pattern. Some subjects maintained moderate or high similarity by the end of extinction (e.g., R7, R9, R10, and R11), whereas others showed little or no correspondence (R8 and R12). The RefLast–RenFirst comparison yielded generally moderate correlations across subjects, with R11 and R12 showing the strongest correspondence, indicating partial recovery of the reinforcement pattern at test. Finally, the ExtFirst–RenFirst comparison was, on average, lower and more variable, and none of the correlations were statistically significant. The ExtLast–RenFirst comparison, which evaluates whether the renewal‐test distribution was more similar to the terminal extinction pattern, was significant only for R11 and did not show a consistent correspondence between the end of extinction and renewal across subjects. Overall, these comparisons suggest that response allocation during renewal was only weakly and inconsistently related to the distributions observed during extinction, and for some subjects it retained features of the spatial pattern established during reinforcement.

**TABLE 2:** Correlation between response distributions for Experiment 2.

Subject	RefLast‐ExtFirst	RefLast‐ExtLast	RefLast‐RenFirst	ExtFirst‐RenFirst	ExtLast‐RenFirst
R7	.975, *p* = .005*	.866, *p* = .058	.700, *p* = .188	.616, *p* = .269	.577, *p* = .308
R8	.800, *p* = .104	.000, *p* = 1	.667, *p* = .219	.205, *p* = .741	.103, *p* = .870
R9	.821, *p* = .089	.821, *p* = .089	.600, *p* = .285	.821, *p* = .089	.820, *p* = .089
R10	.100, *p* = .873	.872, *p* = .054	.667, *p* = .219	.205, *p* = .741	.368, *p* = .542
R11	.975, *p* = .005*	.895, *p* = .040*	.872, *p* = .054	.800, *p* = .104	.975, *p* = .005*
R12	.872, *p* = .054	−.057, *p* = .927	.975, *p* = .005*	.800, *p* = .104	−.224, *p* = .718

*Note*: Calculated with the distribution of responses between levers. RefLast = last session Phase 1. ExtFirst = first session Phase 2. ExtLast = last session Phase 2 with available data points. RenFirst = first session Phase 3.

* = statistically significant correlation. The first value of each cell shows ρ.

**FIGURE 8:**
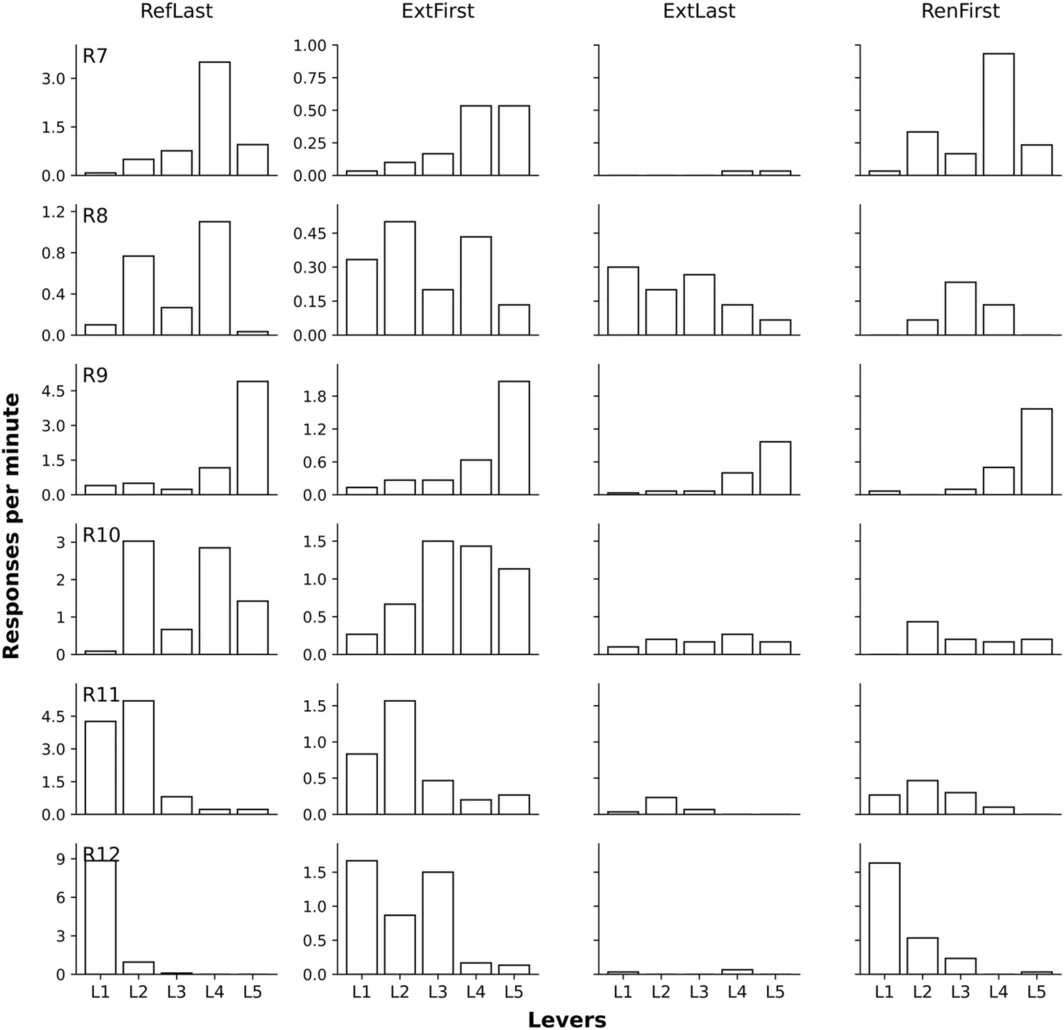
Responses per minute across levers for transition sessions in Experiment 2. RefLast = Last reinforcement session, ExtFirst = First extinction session, ExtLast = Last extinction session, and RenFirst = First renewal test session. The panel for R8 at ExtLast shows responses from the fifth extinction session, as no responses occurred during the last extinction session. Please note the different *y*‐axis between RefLast and the transition sessions under extinction.

Figure 8 shows individual generalization gradients for each rat across the four transition sessions, similar to Figure 4. As mentioned earlier, response distributions during the final session of Phase 1 were heterogeneous across rats. Half of the subjects showed a preference for two response levers. However, R7, R9, and R12 showed a marked preference for one lever. R7 mainly responded at L4, R9 responded at L5, and R12 responded at L1, showing patterns resembling the ones observed in Experiment 1. For these rats, responding during Phase 1 was mostly concentrated on a single lever, with decreasing response rates on adjacent levers, forming a gradient‐like distribution. However, unlike Experiment 1 where gradients were centered on the middle lever, these patterns were observed at different points in the lever array rather than symmetrically around the center.

During the first session of Phase 2, overall response rates decreased, but the distribution of responses still resembled that observed at the end of Phase 1. For five of the six rats (except for R10), most levers that had the highest response rates during reinforcement continued to show the highest levels of responding at the onset of extinction, indicating that the previously established pattern persisted despite the withdrawal of reinforcement and the increase in responding at other levers. By the last session of Phase 2, response rates approached zero across all levers for most rats, except R9 and R1.

During the first session of Phase 3, responding reemerged for all rats and tended to be concentrated on the same lever that had maintained the highest response rates during reinforcement. For five of the six rats (except R8), the lever with the highest response rate during testing corresponded to the lever that had the most responses during Phase 1, suggesting partial recovery of the previously established pattern.

Table 3 shows the mean obtained reinforcement rates on each lever during the last six sessions of Phase 1. Total obtained reinforcement rates ranged from 1.01 to 2.14 reinforcers per minute across rats. All rats except R8 obtained nearly all programmed reinforcers available during these sessions, with R7 and R9 obtaining 299 of 300 reinforcers and R10, R11, and R12 obtaining all 30. R8 obtained 183 of 300 reinforcers, corresponding to a total rate of 1.01 reinforcers per minute. The distribution of obtained reinforcers across levers differed markedly between subjects. Most reinforcers were obtained on L4 for R7 and R8, on L5 for R9, on L2 and L4 for R10, on L1 and L2 for R11, and on L1 for R12. Thus, although reinforcement was available for responses on any lever, each rat contacted a different local distribution of reinforcement across the lever array. Inspection of Figure 8 reveals that reinforcement rates largely determined response rates during the last reinforcement session.

**TABLE 3:** Mean reinforcers per minute during Phase 1 for rats in Experiment 2.

Subject	L1	L2	L3	L4	L5	Total
R7	.03	.04	.22	1.44	.14	1.89*
R8	.01	.20	.17	.60	.02	1.01
R9	.04	.04	.06	.46	1.42	2.05*
R10	.01	1.00	.28	.50	.33	2.14*
R11	.78	.92	.28	.04	.02	2.06*
R12	1.72	.23	.03	.00	.00	1.98*

*Note*: Calculated with the last six Phase 1 sessions.

* = obtained > 99% possible reinforcers.

### Discussion

During this experiment, we observed the development of stable patterns of responding across an array of five levers when responding on each of them was reinforced. After the shift to extinction, we also observed the occurrence of extinction‐induced variability. Then, upon the return to the reinforcement context, we found a renewal effect for all subjects, as responses increased relative to extinction. As in Experiment 1, these effects were predicted and were congruent with the literature (Bouton et al., 2011; Diaz et al., 2019; Nakajima et al., 2000). For most subjects, the correlation between RefLast and RenFirst was higher than the correlation between ExtFirst and RenFirst (R7, R8, R10, R11, and R12), indicating that the spatial distribution observed during testing more closely resembled the pattern established during reinforcement than the redistribution emerging at the onset of extinction. Only R9 showed a different pattern, as it always responded in a graded manner from L5 to L1 throughout the experiment.

These comparisons suggest that for several subjects, the pattern observed during renewal was not fully explainable as a continuation of the extinction‐induced variability observed at the onset of Phase 2 in Context B. Although responding during the renewal test also occurred under extinction in Context A and therefore could include extinction‐induced variability, response allocation appeared to be consistent with reinforcement experienced in Phase 1, even when extinction had altered response distributions.

## GENERAL DISCUSSION

Renewal of lever pressing was found in two experiments with rats as subjects. During Experiment 1, when only responses to the middle lever were reinforced, stable response patterns developed in the shape of a generalization gradient around the reinforced lever, meaning that most nonreinforced responses occurred at levers adjacent to the reinforced one. When extinction was introduced, extinction‐induced variability was observed across response locations. Upon return to the original training context, response patterns transiently resembled the patterns observed during reinforcement and then became more variable, which can be interpreted as an instance of ABA renewal of both response frequency and the distribution of responses maintained by previous reinforcement. Across the three phases, responses were mainly allocated toward the middle of the lever array. During Experiment 2, in which responses to any lever could be reinforced, different response distributions developed for every rat, with half of them developing patterns similar to rats observed in Experiment 1 (e.g., one preferred lever). The extinction procedure induced variability in both experiments. Finally, when exposed to the original training context, responses briefly recovered.

The correlation between the patterns observed during reinforcement and testing was high during Experiment 1 and moderate at best during Experiment 2. One possible reason for this difference is that Phase 1 response patterns were more uniform in Experiment 1, where reinforcement was restricted to the middle lever, than in Experiment 2, where reinforcement could be obtained on any lever and subjects developed more variable distributions. Thus, the spatial pattern established during Phase 1 may have been more clearly defined in Experiment 1, making its partial recovery during testing easier to detect. It should also be noted that although *U* values did not fully return to Phase 1 levels, they reliably shifted in that direction during testing for most subjects during both experiments. It is unlikely that this change in variability simply reflects the passage of time under extinction, as responses were already near zero by the final extinction sessions (see Figures 1 and 5). Instead, the shift in *U* values is more consistent with renewed contextual control by the training context, which partially recovered features of the spatial response distributions established during Phase 1.

The obtained reinforcement rates provide additional support for the interpretation that renewal distributions were constrained by Phase 1 reinforcement histories. In Experiment 1, reinforcement was only available for responses on the middle lever and, for most subjects, at or near the maximum possible rate; accordingly, responding during the renewal test tended to reemerge mainly on the middle lever and, when distributed to other locations, more often on adjacent than distal levers. In Experiment 2, reinforcement was available on any lever but the obtained reinforcement rates were not evenly distributed across the array. Instead, each rat contacted a different local distribution of reinforcement and the levers associated with higher reinforcement rates during Phase 1 generally corresponded to the levers with higher response rates during the renewal test. This relation was not perfect, but it suggests that renewal involved more than an undifferentiated increase in responding. Rather, the spatial allocation of renewed responding appeared to reflect, at least in part, the distribution of reinforcement experienced during training.

Cases of low reinforcement rates were also informative. In Experiment 1, R1 obtained the lowest reinforcement rate during Phase 1 and was also the only subject that did not show an increase in responding during the first renewal‐test session. In Experiment 2, R8 was the only subject with a markedly lower obtained reinforcement rate and was also one of the rats with less renewal and the only rat for which the lever with the highest response rate during testing did not correspond to the lever with the highest response rate during Phase 1. Although these observations are based on individual cases, they are consistent with prior work showing that baseline reinforcement rates can modulate relapse (Berry et al., 2014).

The response patterns established during Experiment 1 allowed for assessment of generalization gradients. However, responding on levers adjacent to the reinforced one was not as frequent as in previous relevant studies. For example, in reports by Escobar and Bruner (2007) and Diaz et al. (2019), the frequency of responses to the levers contiguous to the reinforcement lever were similar. One possible explanation for this difference is the physical arrangement of the levers. Most studies reporting graded effects, like the ones from Escobar and Bruner and Diaz et al., have employed operanda mounted in close proximity to each other or registered responses on a device that allowed continuous measures across axes (e.g., Allan & Zeigler, 1989; Ploog & Zeiler, 1997). In the current experiments, response levers were located 2 cm apart, a distance twice as wide as the levers. Given the spatial separation between response locations, it is possible that the physical arrangement of the apparatus attenuated the magnitude of the observed effects. As noted by Lattal and Oliver (2020), procedural and spatial variables can influence how generalized responding is expressed without necessarily eliminating the underlying process. Thus, although the gradients observed here were not uniformly steep, the systematic allocation of responses to adjacent levers suggests that generalization effects were present and may extend across different apparatus configurations and experimental preparations. For Experiment 2, reinforcement could be contingent on responses on any lever, so the graded patterns would be difficult to qualify. However, for the subjects in that experiment, a preference for one or two levers could be identified around which most responses occurred.

With respect to responding at the transition to Phase 2, findings from the timing literature may provide a useful analogue for understanding how extinction‐induced variability emerges while aspects of prior response organization are still preserved. For example, Sanabria and Killeen (2007) used a peak procedure to analyze how temporal patterns of responding organized during extinction periods. Although their procedure was not identical to ours, it similarly involved transitions from reinforced performance to periods in which responses no longer produced programmed consequences, making it broadly analogous to the shift from our Phase 1 to Phase 2. In their peak‐interval procedure, subjects were first trained under fixed interval (FI) schedules until the characteristic scalloped pattern of responding emerged. Then, subjects were exposed to probe trials (cancellation of programmed FI reinforcement) in which responses were only recorded. Importantly, responding during those unreinforced probes did not become random. Instead, responses remained organized around the previously reinforced temporal value, producing orderly temporal gradients. A similar interpretation may apply to the present data. What remained structured in Sanabria and Killeen's study was the temporal distribution of responding during extinction, whereas what remained structured in the present study, at least during the first few extinction sessions, was the spatial distribution of responding. From this perspective, the persistence of response location during the onset of extinction is consistent with the idea that reinforcement history can continue to constrain behavior even when reinforcement is absent and extinction‐induced variability begins to develop.

In renewal procedures, recurrence is usually interpreted as the recovery of behavior when the organism returns to a context previously correlated with reinforcement. However, the test context usually only resembles the reinforcement context in stimulus dimensions (e.g., visual, olfactory, and auditory features), not in the temporal dimension (see Bouton, 1993, for a discussion of time as a stimulus dimension). In that sense, recurrence may involve elements of stimulus generalization insofar as the contextual conditions present during the test phase are similar but not identical to those present during reinforcement. At the same time, renewed responding may also reflect response generalization, like in the present study, because patterns of responding established during reinforcement can reappear across related response locations. From this perspective, the effects observed in the present experiments may reflect a combination of both processes: the reappearance of response distributions shaped by prior reinforcement and the partial generalization of contextual control across similar stimulus conditions. Disentangling the relative contribution of these processes is difficult within the present design, but future studies could attempt to separate them more explicitly by manipulating contextual similarity or by independently assessing stimulus and response generalization during recurrence tests.

It is conceivable that if the response rates observed during reinforcement are renewed, then the same should be so for measures of response variability. Galizio et al. (2018) have argued that operant variability is consequence sensitive and can recur when conditions that previously supported variability are restored. Our data are consistent with this account in two ways: (a) spatial variability increased when contingencies shifted from reinforcement to extinction, consistent with extinction‐induced variability, and (b) aspects of the Phase 1 spatial patterns often reoccurred when the training context was reintroduced rather than a mere undirected increase in responding. The results from both experiments are congruent with a view of variability as constrained by the interplay between reinforcement and extinction procedures. In this line of reasoning, Neuringer et al. (2001) have argued that the withdrawal of reinforcement will most likely increase variability but that previous performance will reliably be retained.

It is also worth noting that in recurrence procedures, one common strategy has been to include a “control” response to evaluate whether increases in the target response reflect its reinforcement history or simply extinction‐induced variability (Lattal & Oliver, 2020). This approach resembles studies that analyze spatial response distributions, such as the present work and Diaz et al. (2019), who trained rats to press a central lever in an array of seven and observed a generalization gradient that not only formed during training but also reappeared during resurgence, with responses decreasing systematically with distance from the reinforced lever. In our Experiment 1, as in that study, increases in responding at inactive operanda during renewal were not indiscriminate but followed patterns constrained by prior reinforcement history. Similar issues have been raised in human research: Cox et al. (2019) found that responding on control operanda during resurgence tests was often as high as or higher than the target, making interpretation difficult and highlighting the role of preexperimental histories and verbal processes in generating widespread responding. These findings are consistent with Podlesnik and Baum's (2024) analysis, which suggests that variability observed during recurrence should be understood as a broad process that encompasses both topographically similar responses, often described by spatial generalization gradients, and more dissimilar activities. From this perspective, the present results suggest that responding on inactive levers during renewal testing reflects both the reappearance of patterns established by reinforcement history and sampling of infrequent response options that expose the organism to new potential sources of reinforcement.

One feature of the present procedure worth noting, because of its dissimilarity with usual renewal procedures, is that the stimulus light used as response feedback was presented throughout the three phases of the procedure. The light was employed to facilitate discrimination between levers. Its onset correlated with food only in the sense that responses to the middle lever in Experiment 1 were the ones that could meet the reinforcement criteria. During Experiment 2, responses on any lever turned on the light and delivered reinforcement. Therefore, the stimulus light could in principle acquire the function of a conditioned reinforcer. We think this is unlikely. In Eckerman and Lanson's (1969) study, a conditioned reinforcement effect was found for the stimulus light, as it accompanied reinforcement. However, the authors only reported such an effect under continuous reinforcement conditions and there was no mention of its occurrence when subjects were exposed to schedules of reinforcement more similar to those used in the current work (e.g., fixed‐, variable‐, and random‐interval schedules). Furthermore, the light used in Eckerman and Lanson's study had a duration of 3 s. There are reports showing that the efficacy of a conditioned reinforcer is highly dependent on the duration of the stimulus (Dinsmoor et al., 1981). Given that the stimulus light of the current procedure was as brief as the click made by the microswitch operation, we assumed its effect in this respect to be mostly negligible. Both Neuringer (1970) and Gleeson et al. (1989) used brief light flashes of similar duration as response‐produced stimuli in experimental preparations, raising the possibility that such flashes might function as conditioned reinforcers. However, the available evidence suggests that this interpretation is unlikely. Gleeson et al. (1989) directly evaluated this possibility by comparing conditions in which responses could produce or not produce a brief houselight flash and found the effect mostly negligible. Although the flash occasionally produced small and transient increases in responding, its effects were inconsistent and did not account for the overall maintenance of behavior. Moreover, in the current experiments the light was present under identical conditions during reinforcement, extinction, and testing, meaning that it did not uniquely signal reinforcement during the final phase. The effectiveness of a conditioned reinforcer largely depends on its current correlation with primary reinforcement (Kelleher & Gollub, 1962); when that correlation is absent or remains unchanged across phases, its ability to exert differential control over responding is likely to be limited.

Still, the presence of the light throughout the procedure can be viewed as a limitation in the sense that it may have in fact facilitated discrimination among response locations and thereby reduced the likelihood of broad response gradients. It should be noted that contemporary analyses emphasize that generalization is not merely a failure of discrimination, as generalization also reflects the lawful spread of stimulus control across similar situations and allows organisms to respond adaptively to novel environments (Davison & Cowie, 2025). In any case, at present, the precise functional role of the light in the procedure remains unclear. Future experiments might therefore omit this feature or manipulate its presence systematically to evaluate its influence. Until such evidence is available, the present gradients should be interpreted with some caution.

Most of our findings are congruent with previous studies, but there are some results that contrast with previous reports. For some rats in Experiment 2, responses tended to occur at levers closer to the walls of the chamber and thus closer to the food tray on the opposite panel, with the middle lever being mostly ignored. This feature of the data is similar to that reported by Herrnstein (1961), which was interpreted by Eckerman and Lanson (1969) as due to the operanda and food hopper being placed on opposite panels, which forced pigeons to move back and forth. In Eckerman and Lanson's study, which also employed pigeons, this pattern of responding was not found, according to the authors because the food hopper and the response‐recording device were positioned on the same panel. In the present experiment, even when side preference in responding was found, not all rats adhered to such pattern. Contrary to Herrnstein's data in which all subjects responded with an extreme side preference, only two out of the six rats primarily responded at Levers 1 or 5 (see R9 and R12 on Figure 7). One possible explanation for Hernstein's data is that such differences occur because of species‐specific patterns of responding. Perhaps further replication of Hernstein's data with pigeons could shed light on these issues. Overall, our data are consistent with Eckerman and Lanson's findings, even with the levers and food hopper placed on different panels, contributing to the generality of their findings.

Even if the location of the levers and feeder does not systematically affect the development of response patterns toward any specific distribution, after a response pattern is established, response rate seems to follow as a function of the distance from lever to reinforcer. When response patterns were established toward Levers 1 or 5, response rates seemed to stabilize at higher values and tended to recur more (see R9 and R12 on Figure 7) than for rats that established patterns toward levers farther from the sides of the chamber. However, this account of the data is highly speculative, and further replication and experimental manipulation is needed to conclude whether the response location is responsible for the rate or whether a different variable is responsible.

A useful next step in this line of research would be to modify the design in ways that allow stronger experimental control over how spatial patterns are acquired, degraded, and renewed. One promising change would be to manipulate the location of the active operanda more systematically—for example, by training different groups on central versus lateral levers or by alternating trained locations within subjects to determine whether the strong preference observed at some edge levers in Experiment 2 reflect spatial constraints of the apparatus, proximity to the feeder, or the reinforcement history itself. It would also be valuable to arrange repeated renewal probes to determine whether recovery of spatial patterns is transient or stable across repeated context reintroductions. Future designs could parametrically manipulate lever spacing or the availability of alternative responses because these variables may clarify whether renewed response distributions are primarily a product of response generalization, extinction‐induced variability, or interaction with the physical arrangement of the chamber. Finally, future research should also include female rats to evaluate potential sex differences in the magnitude of renewal and in the spatial distribution of responding across levers during extinction and renewal testing.

The present findings may also have translational implications for the analysis of relapse following behavioral treatment. In many applied settings, reductions in problem behavior are achieved through extinction‐based procedures, often combined with differential reinforcement of alternative responses (Kimball et al., 2020; Kimball, Salvetti, et al., 2023). However, relapse frequently occurs when contextual conditions change, such as when an individual moves from a treatment setting to the home environment, when caregivers change, or when treatment integrity is disrupted (Wathen & Podlesnik, 2018). Laboratory renewal procedures have been widely interpreted as models of these contextual transitions because they isolate the role that contextual cues play in controlling the persistence or recurrence of behavior (Bouton et al., 2011; Kelley et al., 2015). From this perspective, the present procedures model situations in which a behavior that has been reduced under extinction may reappear when the organism returns to conditions previously correlated with reinforcement. Importantly, our results suggest that relapse may involve more than a simple increase in the previously reinforced response. Instead, the spatial distribution of responding during renewal was constrained by patterns established during reinforcement. In other words, relapse tended to reproduce aspects of the earlier response organization rather than generating entirely random responding. This may be relevant for understanding relapse of socially significant behavior. For example, in the treatment of severe problem behaviors such as aggression or self‐injury, individuals often display multiple response topographies that belong to the same functional response class (Saini et al., 2018). During treatment, reinforcement contingencies may suppress some responses while leaving others less frequently expressed. When treatment conditions change, these previously suppressed response forms may reappear in patterns influenced by the individual's reinforcement history. Similarly, in functional communication training, relapse may include not only the reoccurrence of the original problem behavior but also shifts toward related responses that had previously occurred at lower rates. In this sense, the spatial distributions analyzed in the present experiments can be interpreted as laboratory analogs of how response classes reorganize during relapse. The lever array provided a controlled way to measure how reinforcement history shapes the distribution of behavior across multiple response options, allowing us to observe how these distributions degrade during extinction and partially reappear during renewal.

More broadly, the results suggest the possibility that relapse processes may involve the recovery of structured response patterns rather than a purely undirected increase in variability. From a translational standpoint, this suggests that interventions designed to mitigate relapse may benefit from considering not only the target behavior but also the broader patterns of responding established during reinforcement. Procedures that promote more distributed or flexible response repertoires during treatment or that arrange reinforcement contingencies across multiple alternative responses may reduce the likelihood that relapse will reproduce a narrow pattern of behavior tied to earlier reinforcement histories. For example, recent work on expanded‐operant treatments shows that training multiple response alternatives so that appropriate behavior can occur in several forms rather than through a single topography may mitigate relapse (Fuhrman et al., 2022; Velasquez et al., 2024). Although this strategy has been studied primarily in the context of resurgence (e.g., serial training of multiple alternative responses), the underlying logic may extend to other relapse phenomena like renewal.

Although the two experiments in the present article are difficult to compare directly because they arranged different reinforcement contingencies, some descriptive differences are noteworthy. In Experiment 2, in which responding on any lever could meet the reinforcement criterion, response rates appeared to be somewhat higher overall, to decline less abruptly during Phase 2, and to show a larger increase relative to baseline during the renewal test than in Experiment 1 (compare Figures 1 and 5). These observations should be interpreted cautiously given that the study was not designed as a direct comparison between contingencies. Still, they raise the possibility that response repertoires established under reinforcement distributed across multiple options may be more persistent under adverse conditions and more likely to reemerge when contextual conditions change. In that limited sense, the present findings may bear some conceptual relation to work on expanded‐operant treatments, which suggests that reinforcing multiple acceptable alternatives can sometimes promote more durable behavior change and reduce exclusive dependence on a single response option. Although our data do not test that possibility directly, nor do they allow strong translational conclusions, they are at least consistent with the idea that behavior supported by a broader reinforcement history may be less vulnerable to complete disruption and more likely to recur when the context shifts.

Although the present experiments were conducted under highly controlled laboratory conditions, the processes they isolate (extinction‐induced variability, contextual control of relapse, and the persistence of reinforcement‐shaped response patterns) may contribute to understanding how behavior reorganizes when treatment conditions change. In summary, our study replicated previous findings in which extinction procedures induced behavioral variability into previously stable response patterns and showed that similar responding tended to renew in most subjects when contexts signaling reinforcement contingencies were reintroduced. These findings extend the generality of recurrence phenomena in operant literature to response location, a usually neglected response dimension. By showing that renewal extends beyond response rates to include generalization gradients, the present results add to the growing body of literature emphasizing the multidimensional nature of relapse.

### AUTHOR CONTRIBUTIONS

All authors contributed to reviewing, commenting, and editing of the manuscript. Additionally: Rodrigo Benavides: Apparatus preparation, data collection, data analysis, and original draft preparation. Rogelio Escobar and Carlos Flores: Conceptualization, funding acquisition and supervision. Brissa Gutierrez: Conceptualization and data analysis.

## CONFLICT OF INTEREST STATEMENT

The authors have no competing interests to declare that are relevant to the content of this article.

## ETHICS APPROVAL

The study followed the guidelines of the institution's ethics committee.

## Data Availability

Data will be made available upon request.
